# Gastrointestinal Ultrasound in Emergency Setting

**DOI:** 10.3390/jcm12030799

**Published:** 2023-01-19

**Authors:** Andrea Boccatonda, Damiano D’Ardes, Viola Tallarico, Susanna Vicari, Elena Bartoli, Gianpaolo Vidili, Maria Teresa Guagnano, Giulio Cocco, Francesco Cipollone, Cosima Schiavone, Esterita Accogli

**Affiliations:** 1 Internal Medicine, Bentivoglio Hospital, AUSL Bologna, Bentivoglio (BO), 40010 Bologna, Italy; 2 Department of Medicine and Aging Science, Institute of “Clinica Medica”, “G. d’Annunzio” University, 66100 Chieti, Italy; 3 Department of Internal Medicine, Bologna University, 40138 Bologna, Italy; 4 Department of Medical, Surgical and Experimental Sciences, University of Sassari, 07100 Sassari, Italy; 5 Internistic Ultrasound Unit, SS Annunziata Hospital, “G. d’Annunzio” University, 66100 Chieti, Italy; 6 Internal Medicine, Centre of Research and Learning in Ultrasound, Maggiore Hospital, 40133 Bologna, Italy

**Keywords:** ultrasound, bowel, emergency, inflammation, abdomen

## Abstract

Acute bowel diseases are responsible for more than one third of subjects who were referred to the emergency department for acute abdominal pain and gastrointestinal evaluation. Gastrointestinal ultrasound (GIUS) is often employed as the first imaging method, with a good diagnostic accuracy in the setting of acute abdomen, and it can be an optimal diagnostic strategy in young females due to the radiation exposure related to X-ray and computed tomography methods. The physician can examine the gastrointestinal system in the area with the greatest tenderness by ultrasound, thus obtaining more information and data on the pathology than the standard physical examination. In this comprehensive review, we have reported the most relevant indications and advantages to using ultrasound in the investigation of abdominal acute pain.

## 1. Introduction

Acute abdomen is a pathological state that requires a fast diagnosis and subsequent specific therapy. Gastrointestinal ultrasound (GIUS) or computed tomography (CT) are commonly employed to evaluate the reason for acute abdominal pain, thereby planning a specific surgical intervention if necessary or avoiding unnecessary surgery [1,2,3].

Acute bowel diseases are responsible for more than one third of subjects who were referred to the emergency department for acute abdominal pain [1,2]. Ultrasound is often employed as the first imaging method to evaluate patients with acute abdominal pain [1,2,4,5]. GIUS is widely available in the emergency setting, inexpensive and non-invasive [3,6]. Ultrasound is characterized by optimal diagnostic accuracy in subjects with abdominal pain and can be the reference method in young females, thus avoiding radiation exposure [3,6]. Physicians can directly examine the gastrointestinal system in the area of the abdomen with the greatest tenderness, thus obtaining more information and data on the disease than the standard physical examination. GIUS can be integrated with clinical data so that the classical physical examination could be more exhaustive if completed with US investigation, which can be easily performed “bedside” and without requiring X-ray radiation.

## 2. Ultrasonographic Anatomy of Gastrointestinal Tract

A general approach to GIUS consists in a systematic scanning of the gastrointestinal tract [7,8,9]. First, a curvilinear low frequency probe is used to obtain a systematic overview; subsequently, a linear high-frequency probe is used for a more detailed examination of the bowel wall [7,8,9,10].

The study of both the large and small intestine usually starts in the right iliac fossa, as the cecum, the ileocecal valve and the terminal ileum are typically located in this area, over the anatomical landmarks represented by the iliopsoas muscle and the right iliac vessels [7,8,9,10].

The small bowel is the most difficult GI tract to examine; the terminal ileum is the only small bowel loop and can be identified due to its fix position in the right iliac fossa and the presence of specific anatomical landmarks. Other segments of the small bowel are difficult to examine, and ultrasound can provide a general overview of the jejunal and ileal loops through a systematic scanning approach that involves parallel lines cranially and caudally through the abdomen and by exerting enough probe pressure to display the deeper part of the abdomen [10,11].

The small bowel is characterized by the presence of valvulae conniventes, which decrease in number and height from the proximal jejunum to the distal ileum, and they are best visualized in fluid-filled bowel loops [10]. The small bowel is usually collapsed after overnight fasting. The normal maximum diameter of small bowel loops is up to 2–2.5 cm [12].

In order to study the large bowel, after the cecum has been identified, the probe is moved in the distal direction following the ascending colon, transverse colon, descending colon, sigmoid colon and the rectum. The colon is located along the peripheral areas of the abdomen, with the ascending and descending colon fixed to the retroperitoneum on the right and left, with a vertical course anterolaterally to the iliopsoas muscle. The location of the transverse and the sigmoid colon can significantly vary due to the different length of the mesocolon. The transverse colon usually has a horizontal course and can often be displayed just behind the gastric antrum, but it may also descend to the lower abdomen in cases of an elongated mesocolon. The sigmoid colon is usually seen into the pelvis, above the urinary bladder, but it can also cross the midline to the right iliac fossa or even extend up to the liver. The rectum may be seen behind the bladder by low-frequency probe [10,13].

The right colon is usually filled with stool and gas, and it allows the visualization of haustration, which gives the colon profile its typical polycyclic appearance in the longitudinal view and limits the bowel wall study to the superficial side. On the contrary, the left colon is often seen in a contracted condition, so that haustration is not usually visible and the bowel wall is better displayed [13].

The normal diameter of the colon is up to 5 cm, but the cecum could be larger in size [13]. The appendix arises from the cecum, about 2–3 cm below the ileocecal valve. Its position is highly variable, ranging between a medial course over the iliopsoas muscle and a medial or lateral elevation, or a retro-cecal course [10]. Although usually considered difficult to study, technical improvements and providers experience allow the display of a normal appendix in more than 50% of subjects [14]. When visualized with a high-frequency probe, the normal appendix appears as a tubular structure with a cul-de-sac on a longitudinal view, and as a target structure with different wall layers on a transverse view. A predominant hyperechoic appearance may result [15,16] if the lumen is filled with gas. In clinical practice, the normal appendiceal diameter measures up to 6 mm [15]. Normal appendiceal walls do not show vascularity on Doppler [17].

**Figure 1:**
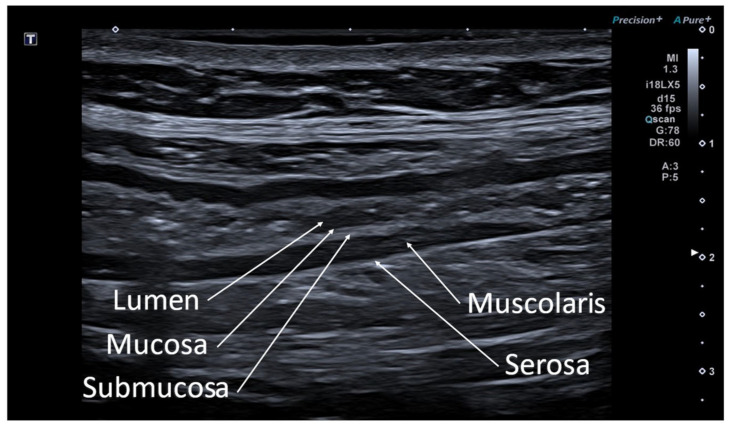
Image obtained with a high frequency linear probe. Longitudinal scan on the parietal stratification of bowel loop: the innermost portion represents the lumen, the first layer (hyperechoic) represents the interface between the mucosa and the lumen, the second layer (hypoechoic) to the mucosa, the third layer (hyperechoic) to the submucosa, the fourth layer (hypoechoic) to the muscolaris propria and fifth layer (hyperechic) to the interface echo between the muscolaris and the serosa.

**Figure 2:**
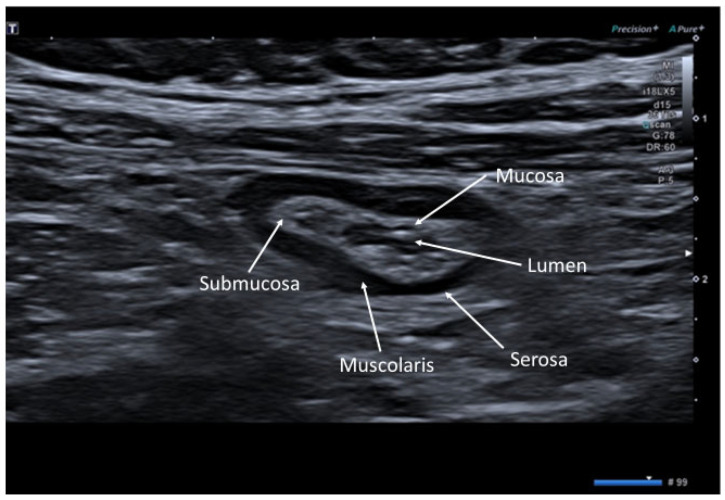
Transverse scan image of a normal bowel loop, in which the succession of hyperechoic and hypoechoic layers is evident.

The bowel wall consists of five sonographic layers when examined with a high frequency transducer (above 5 MHz). The sonographic layers do not exactly correspond to the histological ones, thus representing a combination of interface echoes of the histological layers. Starting from the lumen, the first layer (hyperechoic) is the interface between the mucosa and the lumen, the second layer (hypoechoic) to the mucosa, the third layer (hyperechoic) to the submucosa, the fourth layer (hypoechoic) to the muscolaris propria and the fifth layer (hyperechic) to the interface echo between the muscolaris and the serosa [10] (Figure 1 and Figure 2).

According to the European Federation of Societies for Ultrasound in Medicine and Biology (EFSUMB) Recommendations and Guidelines for Gastrointestinal Ultrasound, bowel wall thickness should be measured perpendicular to the wall, from the interface between the serosa and the proper muscle to the interface between the mucosa and the lumen. A bowel wall thickness less than 2 mm could be considered as normal in a usual filling condition, for both the small and large intestine, with exceptions represented by the duodenal bulb and the rectum [10].

The following sections will analyze the pathological ultrasound findings divided by type of disease.

## 3. Flares and Complication of Inflammatory Bowel Diseases (IBD)

According to the EFSUMB guideline, ultrasound should be employed to evaluate inflammatory bowel diseases (IBD) at first presentation, and to examine its extent, activity and possible complications [18]. GIUS has been demonstrated to be accurate in identifying active IBD, with a sensitivity and specificity of approximately 75% to 90% and 75% to 100% in Crohn’s disease (CD) and 74% to 90% and 93% to 96% in ulcerative colitis, respectively [19,20].

Compared with the alternative options, namely CT and Magnetic Resonance (MR), GIUS has the advantages of being widely available, less expensive, easily repeatable and radiation free [21].

**Figure 3:**
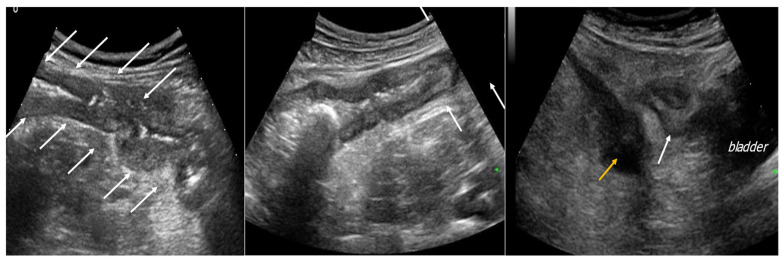
Figures representing Crohn ileitis: the walls are diffusely thickened (white arrows) and a clear distinction between the different parietal layers is lost; the thickening and distortion of the wall can be such as to determine a stenosis of the colon section involved; in the last figure a Crohn ileitis complication is represented [fluid collection (yellow arrow)].

On GIUS, the most frequent pathological findings are bowel wall thickening (Figure 3), changes of the bowel wall echo-pattern, hyperemia of the bowel wall, reduced elasticity and peristalsis, mesenteric fat hypertrophy, enlarged lymph nodes and the presence of abdominal free fluid [22].

The presence of a thickened bowel wall is the most important US finding in CD to detect the disease and is the most frequent index considered in scores used to evaluate IBD activity [19,23]. In the latest studies, the cut-off value most often chosen to define a pathological finding is >3 mm, with a sensitivity and specificity of 89% and 96%, respectively [24,25,26]. When a high specificity is preferred, a bowel wall thickness cut off of >4 mm should be used (sensitivity of 87% and a specificity of 98%) [26].

In the acute phase of CD, the wall thickening is caused by transmural edema: the wall layers are often well defined, and it is possible to identify a typical aspect of a “target sign” on a transverse scan. A few studies have focused on the thickening of each wall layer in CD, finding a prevalent thickening in the submucosal layer, rather than the mucosal and muscolaris layers, in active CD [27,28]. On the other hand, in cases of severe disease, a focal or extensive disruption of the bowel wall layers can be detected due to the presence of deep longitudinal ulcerations [29].

The measurement of bowel wall thickness can be completed by a semi-quantitative evaluation of its vascularity by using color or power Doppler at the level of the most thickened segments, which is correlated with disease activity [18].

**Figure 4:**
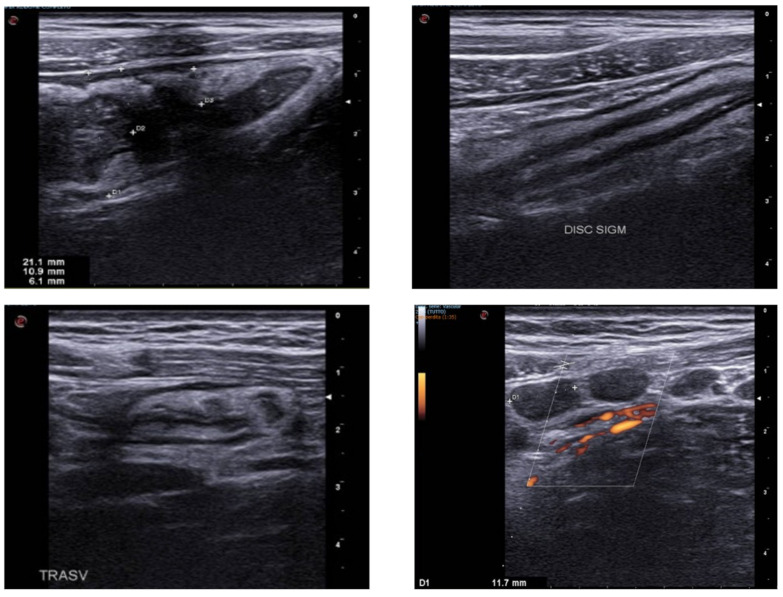
Patient went to the emergency room reporting diffuse abdominal pain for about 10 days associated with diarrhea and traces of blood and mucus, and weight loss (about 10 kg). GIUS showed diffuse wall thickening of the entire colon, particularly at the level of the ileocecal valve (11 mm) and submucosa thickness of 6 mm; multiple lymph nodes were evident in the right iliac area, with a maximum size of 11 mm. The clinical and ultrasound findings are compatible with Crohn’s disease.

In addition to parietal features, GIUS allows the visualization of CD extraintestinal features, such as mesenteric fat hypertrophy, mesenteric loco-regional lymph nodes and abdominal free fluid (Figure 4). Mesenteric fat hypertrophy is a frequent finding in active CD and reflects clinical and biochemical disease activity [30]. Moreover, enlarged mesenteric loco-regional lymph nodes and the presence of a small amount of abdominal free fluid are common, but non-specific, findings in acute CD.

**Figure 5:**
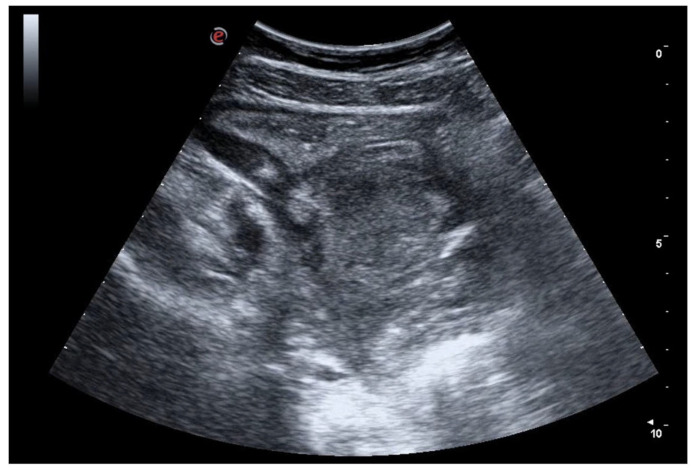
Image obtained with a low frequency convex probe. There is a coarse echogenic formation adjacent to the ileocecal valve which presents a clear wall thickening. Image obtained from a patient with CD who was referred to the emergency room for fever and abdominal pain in the right iliac fossa; final diagnosis of CD acute flare with phlegmon.

GIUS can also detect complications of CD. Strictures are detected on US as a stretch with thickening of the wall and thinning of the lumen: there could be a dilation of the upstream tract, with a possible accumulation of liquid and/or gas. Moreover, fistulas can be observed as hypoechoic tract connecting two loops to other structures, such as the skin or bladder, and are usually characterized by vascularized walls. Abscesses could be also detected: they are represented by hypo-anechoic lesions with liquid content. Phlegmon could also be found in patients with CD, and it is characterized by hypoechoic lesions with faded edges in the absence of colliquation (Figure 5).

In that setting, CEUS could represent a valid tool for a more specific diagnosis. Eventually, GIUS can also detect signs of perforation, characterized by the presence of free air in the subdiaphragmatic region or intraperitoneal liquid mixed with air bubbles.

**Figure 6:**
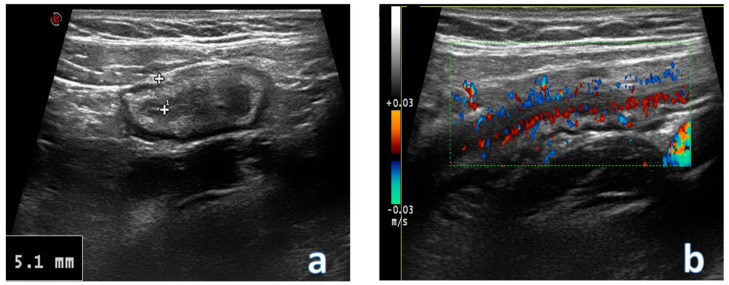
Images obtained with a high frequency linear probe. The image (**a**) shows a transversal scan of a section of colon with thickened walls (ulcerative colitis diagnosis); there is a hypertrophic feature of the submucosa layer. In image (**b**) the color function is inserted, which shows a hypervascularization of the wall, compatible with acute ulcerative colitis flare.

Concerning ulcerative colitis, the disease typically involves the colic walls, which appear continuously and concentrically thickened (over 4 mm) with an absence of haustrations (Figure 6). The most relevant complication is the toxic mega colon that appear as an abnormal dilation of the large bowel lumen (more than 6 cm) coexisting with a reduced wall thickness and dilation and liquid distension in the intestinal loops.

## 4. Acute Appendicitis

Acute appendicitis is one of the most frequent abdominal emergencies worldwide, with an incidence of approximately 100 per 100,000 person-years and with a lifetime prevalence of 7–8% [31,32]. The classical presentation of acute appendicitis includes right iliac fossa abdominal pain (often migratory) with localized tenderness, fever, anorexia, nausea and vomiting. However, early features of appendicitis may be subtle, and elderly and frail patients can present with nonclassical or non-specific features [33].

Despite its high incidence, the diagnostic approach to acute appendicitis is still debated: some guidelines employ scoring systems, others suggest physician clinical assessment alone, and some guidelines include standardized imaging [34,35,36]. As several studies have demonstrated a marked reduction in the negative laparotomy rate with the use of abdominal ultrasound before surgery [37,38], recent guidelines have recommend GIUS to be routinely used in every patient with suspected appendicitis [2,35,39]. Indeed, the sensitivity and specificity of GIUS performed by experienced providers are above 90%, equivalent to CT or MR, with the advantage of being widely available, noninvasive and without ionizing radiation [40,41].

The role of GIUS in cases of suspected acute appendicitis is to confirm the diagnosis or to rule it out by demonstrating a normal appendix over its entire length, and to exclude an alternative cause of abdominal pain [2]. Moreover, GIUS can differentiate between uncomplicated and complicated appendicitis, as non-chirurgic management of uncomplicated appendicitis is recommended [42].

In cases of suspected appendicitis, it is recommended to search the inflamed appendix at the point of the greatest abdominal pain, pointed out by the patient, using graded compression [43]. Alternatively, a systematic approach involving the localization of terminal ileum, cecum and the origin of the appendix, 2–3 cm below the caecum, can be used [2].

**Figure 7:**
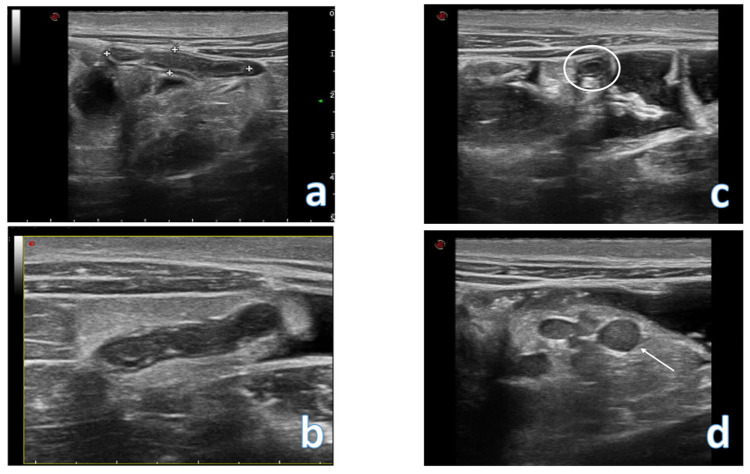
Images of a case of appendicitis. In figures (**a**,**b**) a longitudinal scan of the appendix is evident, which presents thickened walls and a hypoechoic appearance; compression with the probe does not allow the lumen to collapse and this maneuver evokes elective pain; In figure (**c**) the appendix is visualized in cross scan (white circle); In figure (**d**) multiple lymph nodes are evident in the right iliac fossa (white arrow).

In patients with acute appendicitis, the most accurate GIUS finding is a maximum outer diameter of >6 mm, with a sensitivity and specificity of 98% [15,44]. Additional findings consistent with the diagnosis of acute appendicitis are the incompressibility of the appendix [45], maximal tenderness over the appendix [46], the presence of large fecaliths [47,48] and an increased wall vascularity in color Doppler [49] (Figure 7). This sign is transiently detectable, and hypervascularity disappears in complicated appendicitis due to the ischemic changes in the appendix walls: therefore, increased wall vascularity rules-in appendicitis, but absent vascularity does not exclude it [17,50].

Secondary US signs related to the inflammation of the surrounding tissues are described, such as peri-appendiceal fluid, mesenteric lymphadenopathy and hyperechoic peri-appendiceal tissue (mesenteric fat hypertrophy) [45,47,51].

In clinical practice, only the combination of different GIUS signs allows the diagnosis of acute appendicitis to be reached [45,51]. The detection of an appendix with thickened walls and hyperechoic peri-appendiceal tissue over the area of the greatest pain are the most relevant criteria in the confirmation of the diagnosis [52], while mesenteric lymphadenopathy and the color-doppler evaluation of the appendix are not specific signs and could be demonstrated in several conditions [2,45,51].

The importance of complicated appendicitis (namely gangrenous appendicitis and perforated appendicitis) in the identification of signs relies on the consequences for its management, as the confirmation of complicated appendicitis usually excludes a conservative treatment [53].

The loss of stratification of the appendix wall, and particularly the loss of the echogenic submucosal layer, is the main GIUS sign of gangrenous appendicitis, which can be associated with the lack of vascularization on color Doppler [2,54].

Extraluminal gas, complex fluid collection (peri-cecal abscess) and extraluminal fecaliths are GIUS signs associated with perforation [45,51]. On the contrary, intraluminal fecaliths are not a sign of acute appendicitis with complications, but they are related to a higher risk of perforation and recurrence [2,55].

## 5. Acute Diverticulitis

Colonic diverticula are a common finding in the general population, with a prevalence of <5% under the age of 40 and >65% over the age of 80 [56]. Acute diverticulitis occurs in around 5% of subjects affected by diverticulosis, often with recurrent flares [57].

The clinical presentation of acute diverticulitis includes prolonged lower abdominal pain (usually on the left side) or abdominal tenderness, changes in bowel movements, fever and increased laboratory inflammatory markers [58]. However, the clinical diagnosis of acute diverticulitis usually lacks accuracy; therefore, additional imaging is recommended by most of guidelines [2,59]. Moreover, imaging is a useful tool for early risk stratification, enabling the identification of complications such as abscesses, perforations, fistule or stenosis [2].

Regarding the preferred imaging technique, CT scan is sometimes still considered the gold standard [60]. Recently, due to the comparable sensitivity and specificity of GIUS and CT in diagnosing acute diverticulitis [46,61], provided that GIUS is performed by an expert investigator, other guidelines suggest a step-up approach with GIUS being the first diagnostic method, and that CT should be performed only in the case of a non-definitive ultrasound report or in the case of high clinical suspicion for an acute disease despite negative GIUS [2,58,61]. CT has particular advantages for diseases located in the distal sigmoid or suboptimal US scanning conditions, such as in obese patients [2]. In cases of complicated diseases, CT is usually needed, as it offers a more comprehensive evaluation and can guide therapeutic interventions [58].

Once the sigmoid colon has been identified ventral to the left iliac artery, it can be searched distally and proximally to the descending colon. In cases of suspected acute diverticulitis, an alternative approach consists in starting the exam at the point of maximum tenderness, using the graded compression technique [2,62].

**Figure 8:**
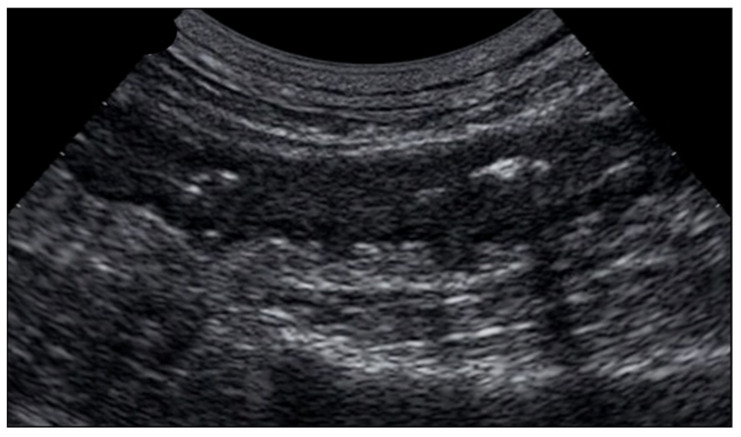
The image represents a section of the descending colon with diffusely thickened and hypoechoic walls; in particular, there are multiple outpourings of the wall, compatible with diverticula.

Diverticula in the colon are visualized on ultrasound as outpouchings of the wall that normally contain echogenic material, represented by gaseous interfaces, feces or fecaliths, and characterized by acoustic shadowing [63] (Figure 8). In sigmoid colon diverticulosis, a slight thickening of the muscularis propria due to the hypertrophy of the circular smooth muscle is often present [56].

The diagnostic GIUS criteria for acute diverticulitis include at least two of the following: short-segmental bowel wall thickening (>5 mm), the presence of an inflamed diverticulum in the wall-thickened area and pericolic tissue changes. Those signs are usually present at the area of the greatest pain, induced by probe compression [2,56,63].

**Figure 9:**
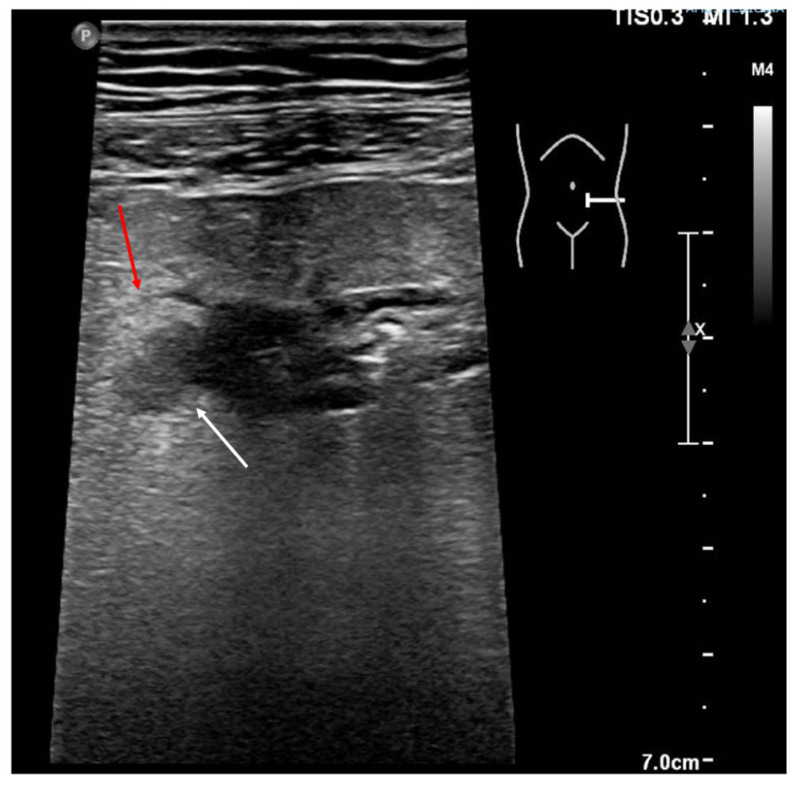
Image obtained by a linear probe. There is evidence of bowel wall thickening and parietal outpouring (white arrow) of a section of the descending colon, compatible with a diverticulum. The hypoechoic formation extends into the surrounding fatty tissue, which appears hyperechoic (red arrow). The findings appear indicative of acute diverticulitis.

**Figure 10:**
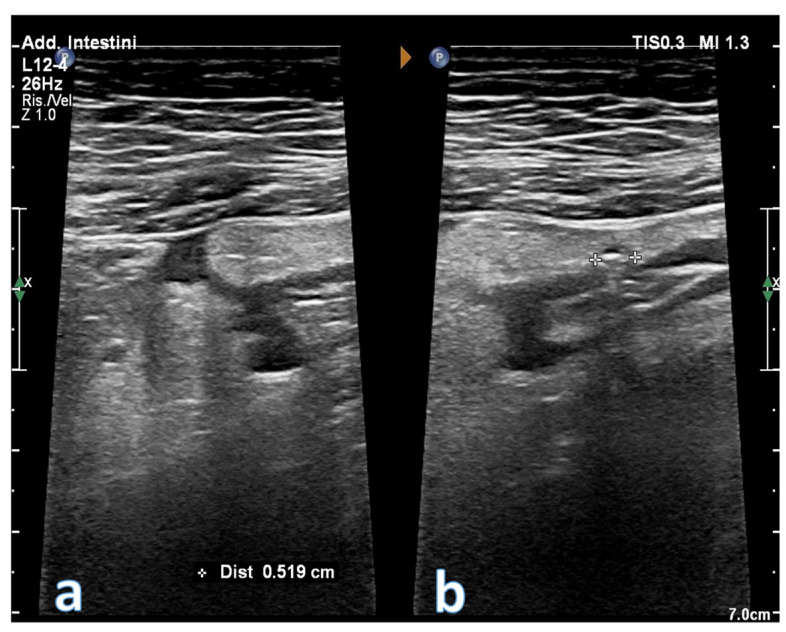
Images obtained with a high frequency linear probe. Sigmoid tract with thickened walls and evidence of a diverticular formation of the wall with an aerial artifact inside it (**b**). The peri-sigmoid adipose tissue is thickened and hyperechoic. In image (**a**) an anechoic area is also visible which from the sigmoid wall moves towards the surrounding adipose tissue, suggestive for abscess area.

The aspect of an inflamed diverticulum may range between hypoechoic (37% of cases), predominantly hyperechoic (4%), hyperechoic with surrounding hypoechoic rim (41%) and hyperechoic with acoustic shadowing (18%) [13] (Figure 8). Pericolic tissue changes in uncomplicated acute diverticulitis are mainly represented by mesenteric hypertrophy, which appears hyperechoic and non-compressible [2] (Figure 9 and Figure 10). Enlarged mesenteric lymph nodes may be found [63]. A Color Doppler examination of the thickened segment may provide additional information for distinguishing between ischemic and non-ischemic causes of bowel wall thickening [64].

Abscess, fistulas, perforation and stenosis are common complications of acute diverticulitis. The sonographic appearance of diverticular abscesses is highly variable: the most typical aspect consists of a collection of hypoechoic fluid, but sometimes they show a prevalent hyperechoic aspect owing to echogenic solid or gaseous content [2]. CEUS is an optimal tool for differentiating hypoechoic abscesses from abdominal phlegmons (both present as hypoechoic masses), as phlegmons show intra-lesional contrast-enhancement, while only partial enhancement is observed in abscesses [65].

Fistulas may extend to the nearby bowel loop, urinary bladder or uterus. They can be identified as hypoechoic bands with or without central gas bubbles [63]. The presence of gaseous artifacts in the bladder suggests a sigmoid-vesical fistula [63].

The typical sign of perforation is the presence of gas bubbles outside the bowel loops. In cases of free perforation, a major complication of acute diverticulitis, the free air is displayed as a hyperechoic line along the hepatic surface or the peritoneal line [2,63].

## 6. Bowel Obstruction

Bowel obstruction is a common cause of acute abdominal pain leading to emergency department admission. Small bowel obstruction (SBO) accounts for approximately 80% of cases of mechanical intestinal obstruction [66]. SBO can be functional (ileus) or mechanical. The main cause of mechanical intestinal obstruction are adhesions from previous surgery, while CD, tumors, hernias and volvulus are rarer causes [67,68]. Large bowel obstruction (LBO) is 4–5 folds less common and is, in most cases, caused by colonic tumors [69].

The clinical presentation of bowel obstruction depends on the location and the cause of the obstruction, and often includes abdominal pain, nausea, vomit, the abolished passage of flatus and/or stools and abdominal distension. Indeed, clinical presentation is non-specific, and imaging is mandatory to confirm the diagnosis and to distinguish between mechanical and functional SBO, to identify the site and cause of the obstruction, and to assess the risk of complications (intestinal ischemia) and the appropriateness of non-surgical management [1,70].

Among the available imaging techniques, GIUS shows a similar accuracy than CT (sensitivity of 87%, specificity of 81%), and higher than X-ray in the detection of SBO [71].

A recent meta-analysis by Lin et al. evaluating fifteen studies showed that GIUS display a sensitivity and specificity of 92% and 93%, respectively, in the diagnosis of SBO [72]. Indeed, given the well-known advantages, ultrasound is recommended as the first screening method to detect the presence of bowel obstruction [1]. Otherwise, the reliability of ultrasound to ascertain the site and cause of the obstruction is lower than CT; therefore, it may be appropriate to combine both of techniques [70].

**Figure 11:**
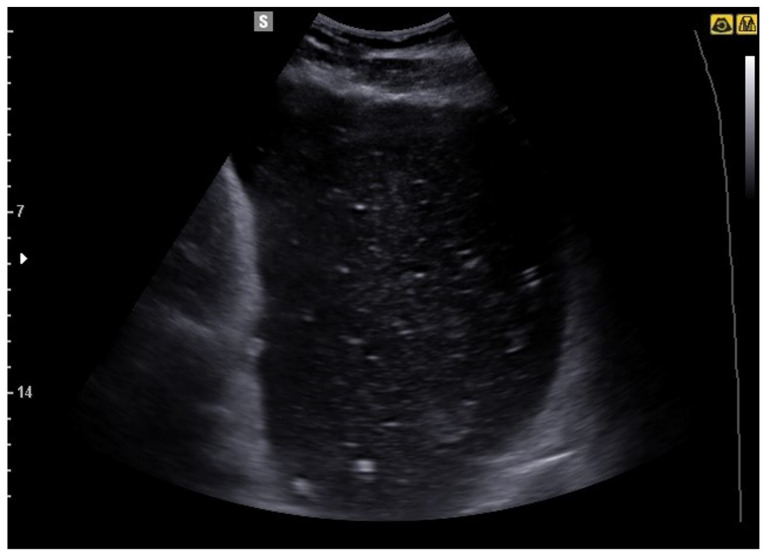
Image obtained with convex probe. There is an overdistension of the stomach with clear evidence of echoes inside it from ingestion. Final diagnosis of duodenal cancer with overdistention of the stomach upstream.

A three-step approach is suggested to establish the presence of bowel obstruction and define the pathological segments. Firstly, the epigastrium should be scanned to assess the stomach; a trans-lienal view can be added to display the gastric fundus [6]. In cases of gastric or duodenal obstruction, only the stomach is outstretched (Figure 11). Moreover, this technique is useful for evaluating the need for nasogastric tube placement. Afterwards, a left mid-abdominal view enables the evaluation of the jejunum and descending colon. Thirdly, a right lower abdomen view enables the evaluation of the ileocecal junction [1].

### 6.1. GIUS Signs of SBO

**Figure 12:**
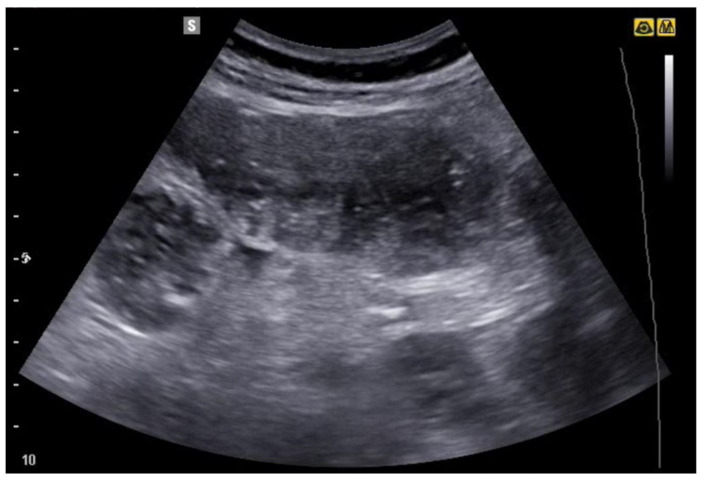
Image obtained with convex probe. Dilated loops of small intestine, with fecal material inside and thinned walls. Diagnosis of small bowel obstruction due to adhesions.

SBO can be diagnosed in the presence of dilated (up to 2.5–3 cm) bowel loops detected to be at least 10 cm in length [70]. The appearance of the bowel content may range between corpuscolated (more frequent in recent or sub-occlusive forms) and anechoic (more frequent in prolonged forms) [70] (Figure 12).

Increased peristaltic “to-and-fro” movement of the bowel loops (which tends to decrease in advanced forms) is a typical finding of acute mechanical obstruction, with the visualization of collapsed loops beyond the stenotic tract [1]. The identification of the transition point, between the dilated proximal and the collapsed distal loops, allow to define the site and cause of the occlusion: the site of obstruction is the distal/terminal ileum if it is in the right iliac fossa and lower quadrants, while it is the jejunum/proximal ileum if it is in the upper quadrants and left hypochondrium. Regarding the cause of the occlusion, the loss of normal visceral sliding is suggestive of abdominal wall adhesions, while deep visceral adhesions are difficult to identify [73]. Other rarer causes of occlusion, such as intussusception, tumors, foreign bodies and external hernias, can be diagnosed due to specific sonographic appearance [1].

It is relevant to consider the alteration of the peristalsis as a criterion for the diagnosis of mechanical ileus [74], which could be reduced, ineffective or absent (paying attention to false movement due to diaphragm and not to intestinal walls). Moreover, it is relevant to point out that the diameter is not an absolute criterion to diagnose bowel obstruction during an early phase of the disease, and other signs must be considered (for example, fluid-filled and hyperkinetic bowel loops with plicar hyper-representation [75].

**Figure 13:**
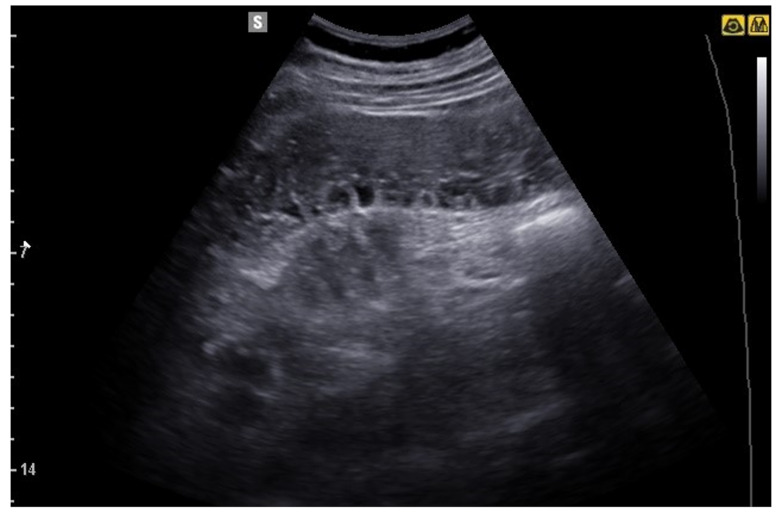
Image obtained with convex probe. Dilated small bowel loops, with evidence of Kerckring valves also called valvulae conniventes (“keyboard sign”), and corpuscular material inside. In live examination there is a to-and-fro movement. Diagnosis: small bowel obstruction due to adhesions.

If the obstruction persists, it could enhance the endoluminal pressure with an increase in the liquid content between the mesenteric recesses (‘sign of the thong’) [76] and then in the abdominal cavity. The detection of free fluid is linked to bowel wall vascular changes [77,78], such as the thickening of valvulae conniventes and the disruption of wall stratification, with normal thickness at 1–3 mm, wall thickening >3 mm, thinned walls <1 mm [18,70]. When the obstruction induces a great accumulation of fluids dilating the loops, it is possible to detect the Kerckring valves, also called valvulae conniventes (“keyboard sign”) [79] (Figure 13; see Supplementary Materials Video S1).

### 6.2. GIUS Signs of LBO

LBO is detected in ultrasound as a clear transition from a dilated (>4.5 cm) to a non-dilated part of the colon, often with liquid content in the right colon and solid stools in the left colon. Otherwise, it is often not possible to measure the diameter and to obtain a wide visualization due to the presence of gas in the obstructed colon.

## 7. Gastrointestinal Perforation

Gastrointestinal perforation is a rare cause of acute abdominal pain in emergency departments [80]. Peptic ulcer, diverticulitis, ischemic bowel disease, blunt or penetrating trauma, iatrogenic factors, foreign body or neoplasm are major determinants of GI perforation [81].

The sudden onset of severe abdominal pain is the main symptom, but patients can report only mild symptoms, depending on the perforation site and the amount of leakage of the intestinal contents [80].

The detection of free gas in the abdominal cavity (pneumoperitoneum) is the most relevant finding suggestive of gastrointestinal perforation [82]. Convex probes are often used, but linear probes can more clearly detect small gas bubbles and allow to differentiate intraluminal vs. extraluminal gas [83]. Some data suggest an examination protocol based on scans in the epigastrium, right and left hypochondrium, umbilical area in the supine position and right hypochondrium in the left lateral position; that protocol seems to be better than a “2-scan fast exam” based on epigastrium and right hypochondrium scans [80].

US signs of pneumoperitoneum should be searched between the liver and the abdominal wall, and they are represented by an enhancement of the peritoneal stripe and hyperechoic lines with reverberation and ringdown artifacts (“dirty shadowing”). Air artifacts (gas) movement, according to patient position (shifting phenomenon), is very suggestive of pneumoperitoneum [84]. Different maneuvers have been proposed to detect those signs; one protocol is based on shifting the patient from the supine to the left lateral position, and to show air artifacts movement [85] (see Supplementary Materials Video S2). When air artifacts (gas) hide the left liver lobe, the application and release of pressure by the probe displaces the artifacts, and the liver appears and disappears [85]. The scissors maneuver is based on the application and subsequent release of pressure on the abdominal wall by the caudal part of the linear probe [86]. Bowel wall thickening, bowel dilatation, free fluid (with fibrinoid septa) and changes in the mesenteric fat are additional indirect signs of perforation [83,85].

Ultrasound is characterized by a better sensitivity than abdominal X-ray (86% compared with 76%) for the diagnosis of pneumoperitoneum [87]. GIUS is characterized by a better diagnostic accuracy than upright chest and left lateral decubitus abdominal X-rays, which cannot detect perforations in 20% to 62% of cases [88,89].

## 8. Ischemic Bowel Disease

**Figure 14:**
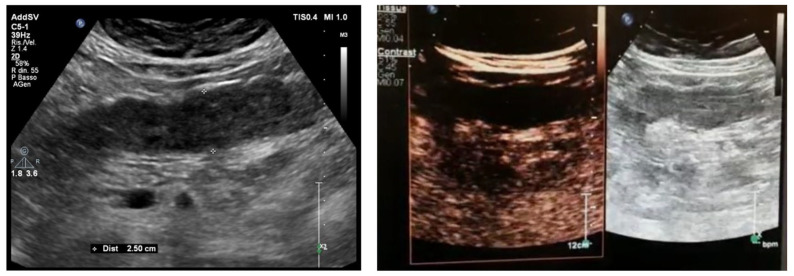
A dilated loop of the small intestine is visualized, with complete loss of wall stratification and diffuse hypoechoic appearance of the wall. In live examination, peristalsis was abolished at the level of that bowel tract. Upon CEUS, there was no parietal perfusion. Image obtained from a patient with newly diagnosed atrial fibrillation and diagnosis of intestinal ischemia (embolic nature).

Ischemic colitis can develop as a consequence of arterial or venous embolism or thrombosis [90,91,92]. Low cardiac output and vasculitis are specific diseases related to bowel embolism or thrombosis [93]. In cases of acute mesenteric ischemia (AMI), the patient usually refers to abrupt severe abdominal pain [91,94]. Other clinical features of AMI are nausea, vomiting, gastrointestinal bleeding, leukocytosis and acidosis [95]. Hematochezia, diarrhea and/or abdominal pain are symptoms suggestive of ischemic colitis. Abdomen CT with a contrast medium is the reference method to study patients with intestinal ischemia [94]. In US examination, bowel wall thickening, decreased peristalsis and increased intraluminal secretions can be detected [96]. Color doppler may reveal the absence of flow in cases of vessel obstruction near the origin of the superior mesenteric artery [97]. Otherwise, the detection of color-flow in the proximal part of the vessel does not exclude the occlusion of distal portions of the mesenteric vessels. CEUS can be useful in evaluating the patency of the vessel and the absence of the vascularization of the bowel wall [98] (Figure 14). In a late phase of the disease, the lumen is filled with fluid, the bowel wall is thickened, extraluminal fluid can be present and peristalsis is abolished [99]. The detection of pneumatosis intestinalis and gas in the portal vein are complications of bowel infarction in advanced stages [99].

### 8.1. Non-Occlusive Mesenteric Ischemia

Non-occlusive mesenteric ischemia (NOMI) is most commonly caused by primary mesenteric arterial vasoconstriction. Most cases involve the spasm of the branches of the superior mesenteric artery (SMA), which supply the small intestine and proximal colon. In GIUS, the walls of the ischemic colon are thickened, hypoechoic and with altered stratification. In the acute phase, there are few color doppler signals on the wall, while moderate hypervascularization can be present after the reperfusion of the bowel. In CEUS, the pathological bowel segments are poorly enhanced and thus perfused [100].

### 8.2. Acute Venous Mesenteric Ischemia

Superior mesenteric vein occlusion can induce edema and the bleeding of the mucosa. The homogeneously hypoechoic thickening of the wall, decreased peristalsis, intraluminal secretions and peri-enteric free fluid are typical sonographic findings [99]. Bowel wall thickening is more evident in cases of mesenteric vein occlusion than the occlusion of the mesenteric arteries [101].

### 8.3. Ischemic Colitis

Ischemic colitis (IC) is the most frequent form of bowel ischemia and the second most frequent reason for lower gastrointestinal bleeding. IC may be characterized by two clinical presentations: a gangrenous form with transmural necrosis and worse prognosis, and a transient form with a segmental involvement of the mucosa or submucosa only, which is usually reversible [102].

In ultrasound, the bowel walls are thickened circumferentially and with a hypoechoic echostructure and a loss of mural stratification. Flow signals of the bowel wall can be not present or decreased during the early phase [103]. Those sonographic features display a high positive predictive value (90%) for IC diagnosis [104]. In cases of reversible disease and reperfusion, color-doppler signals can be detected; CEUS can be employed to better evaluate the perfusion [105]. Changes in the peri-enteric fat have been related to transmural necrosis [103,106]. IC is often characterized by a fast healing of the pathological areas on the mucosa in the absence of transmural necrosis. Ultrasound and CT are characterized by the same accuracy to diagnose IC, but pneumatosis is better detected by CT than ultrasound [101,107].

## 9. Conclusions

Acute bowel diseases are often responsible for acute abdominal pain in the emergency setting. GIUS is widely available, easily accessible, low-cost, noninvasive and is characterized by good diagnostic accuracy in patients with acute abdomen. Therefore, GIUS represents a “bed-side” and non-X ray technique that could complete physical examination, giving the physicians the opportunity to integrate clinical data, signs, symptoms and immediately available imaging. More studies are needed to better define the role of GIUS in the emergency setting and to compare sonographic investigations with X-ray techniques in terms of availability, diagnostic accuracy, costs, and the time it takes to reach the diagnosis.

## Supplementary Materials

The following supporting information can be downloaded at: https://www.mdpi.com/article/10.3390/jcm12030799/s1, Video S1: Examination performed by high-frequency linear probe, with right coronal scan of the patient in left lateral decubitus position. Performing a longitudinal scan, in the first part of the video the ribs are evident, with the pleural line and the A lines; when the thoracic-abdominal passage is scanned, the A lines are evident in the right part of the image, the diaphragm is evident in the upper part of the image (hypoechoic structure with internal hyperechoic line) and below it the liver parenchyma is not visualized but further artefacts of an aerial type are evident (similar A lines); those are sign of pneumoperitoneum; Video S2: Small bowel obstruction due to adhesions. In live examination there is a to-and-fro movement suggestive for mechanical obstruction.
